# Granulosa Cell-Secreted KITL Is Involved in Maintaining Zinc Homeostasis in the Oocytes of Neonatal Mouse Ovaries

**DOI:** 10.3390/antiox14111345

**Published:** 2025-11-10

**Authors:** Yan Du, Lincheng Han, Hongwei Wei, Xiaodan Zhang, Wenbo Zhang, Yashuang Weng, Weiyong Wang, Luchun Zhang, Sihui He, Meijia Zhang, Jingjie Li

**Affiliations:** 1 The Innovation Centre of Ministry of Education for Development and Diseases, School of Medicine, South China University of Technology, Guangzhou 510006, China; 18763811882@163.com (Y.D.); nonohan97@163.com (L.H.); 18390237338@163.com (H.W.); zxdscut@scut.edu.cn (X.Z.); 202410192028@mail.scut.edu.cn (W.Z.); ysweng21@163.com (Y.W.); wangwy@kust.edu.cn (W.W.); 15666939712@163.com (L.Z.); 15626459393@163.com (S.H.); 2 Reproductive Medicine Center, The Sixth Affiliated Hospital, Sun Yat-sen University, Guangzhou 510080, China

**Keywords:** granulosa cells, oocytes, zinc homeostasis, KITL, KIT, oxidative stress

## Abstract

Proto-oncogenic receptor tyrosine kinase (KIT) ligand (KITL) secreted by granulosa cells and its receptor KIT on oocytes are crucial for primordial follicle formation and activation, and follicular development. In the present study, ZnSO_4_ decreased the number of primordial and growing follicles in cultured neonatal mouse ovaries when KITL-KIT signaling was inhibited by ISCK03. ZnSO_4_ also significantly increased the mRNA and protein levels of Zrt/Irt-like protein 6 (ZIP6, a zinc importer) and zinc levels in the oocytes of cultured neonatal mouse ovaries in the presence of ISCK03, suggesting that the increase in ZIP6 levels results in zinc overload in the oocytes of cultured neonatal mouse ovaries. Further experiments indicated that zinc overload resulted in oocyte apoptosis in cultured neonatal mouse ovaries via oxidative stress-driven dual mechanisms: irreversible DNA damage in the nucleus and autophagic flux blockade in the cytoplasm of oocytes. Moreover, the intraperitoneal injection of ZnSO_4_ and ISCK03 significantly increased ZIP6 expression, DNA damage, autophagic flux blockade, and apoptosis of oocytes in neonatal mice. Taken together, these findings indicate that granulosa cell-secreted KITL is involved in maintaining zinc homeostasis in the oocytes of neonatal mouse ovaries. This study not only reveals a novel function of granulosa cells in supporting oocyte homeostasis, but also provides a theoretical basis for identifying individuals susceptible to zinc dyshomeostasis caused by the impaired KITL-KIT signaling.

## 1. Introduction

In mammals, primordial germ cells undergo mitosis to form germ cell nests, which are subsequently broken down to establish the nonrenewable primordial follicle pool [1]. Only a few primordial follicles are recruited into the growing follicle pool in each wave of recruitment, and most of them remain in a dormant state to maintain female reproductive lifespan [2]. As the basic functional unit of the ovary, ovarian follicles consist of oocytes and their surrounding granulosa cells [3]. The communication between somatic/granulosa cells and oocytes is essential for primordial follicle formation and activation, and follicular development [4]. One of the important ligand-receptor systems is proto-oncogenic receptor tyrosine kinase (KIT) ligand (KITL) secreted by somatic/granulosa cells and its receptor KIT, expressed on oocytes [5,6]. KITL participates in primordial follicle formation by activating the Janus kinase (JAK) signaling pathway to dissociate germ cell cysts [7], primordial follicle activation by activating the phosphoinositide 3-kinase/protein kinase B (PI3K/Akt) signaling pathway [8,9], and follicular development by activating the Sma- and Mad-related protein (SMAD) signaling pathway [10,11]. ISCK03, a specific KIT inhibitor, binds to KIT and inhibits its tyrosine kinase activity and autophosphorylation, thereby reducing the phosphorylation levels of key downstream signaling molecules such as Akt and ERK1/2 [12]. Therefore, ISCK03 is widely used to study the role of the KITL-KIT signaling in various physiological and pathological processes [13,14].

As a trace element, zinc exists as a divalent cation (Zn^2+^) and plays various roles [15]: as a structural component of proteins and nucleic acids for their folding [16], as a catalytic center for enzyme activity [17], and as an intracellular second messenger for regulating receptors and target proteins [18]. Zinc homeostasis is regulated by the coordinated action of Zrt/Irt-like proteins (ZIPs, zinc importers), zinc transporters (ZnTs, zinc exporters), and metallothioneins (MTs, zinc-binding proteins) [19]. It has been reported that ZIP6- and ZIP10-promoted zinc influx is required for mouse oocyte meiotic maturation [20,21]. ZIP9 participates in oocyte maturation by promoting extracellular zinc transport into the cytoplasm [22]. ZnT3 can transport zinc into oocyte vesicles, providing a material basis for the generation of zinc sparks during fertilization [23]. ZnT9 enhances the antioxidant capacity of oocytes in antral follicles by transporting zinc from the mitochondrial matrix to the cytoplasm [24]. Furthermore, MT1 and MT2 participate in follicular development by chelating excess zinc in oocyte cytoplasm to prevent mitochondrial oxidative stress, and their expression is enhanced by metal regulatory transcription factor 1 (MTF1) [19,25]. These factors coordinately maintain zinc homeostasis in oocytes during follicular development. However, zinc overload in oocytes leads to aberrant meiotic progression by disrupting spindle assembly [26], and finally oocyte apoptosis due to oxidative stress and DNA damage [27]. Therefore, the precise regulation of intracellular zinc levels is crucial for maintaining the growth and development of oocytes.

Our previous studies demonstrated that ZnSO_4_ promoted primordial follicle activation through the mammalian target of rapamycin (mTOR)-KITL/KIT-PI3K/Akt signaling pathway [28]. Here, we found that ZnSO_4_ induced zinc overload in the oocytes of neonatal mouse ovaries in the presence of the KITL-KIT signaling inhibitor ISCK03, ultimately resulting in oocyte apoptosis. This mechanism was involved in the overexpression of ZIP6 in the oocytes. Our findings reveal the critical role of KITL-KIT signaling in maintaining zinc homeostasis in the oocytes of neonatal mouse ovaries.

## 2. Materials and Methods

### 2.1. Animals and Chemicals

Adult ICR mice (2 months old) were purchased from the Guangdong Medical Laboratory Animal Center (Guangzhou, China). Controlled conditions were used to raise the animals: a temperature controlled at 22 °C with a 2 °C variation, a humidity of 50–70%, and a cycle of 12/12 h light/dark. These mice were provided with unrestricted access to food and water, and were mated in a male/female ratio of 1:1. The day of neonatal mouse birth was deemed 0.5 days postpartum (dpp). Three-days-postpartum female mice were used for intraperitoneal injection or ovary culture. The Animal Care and Use Committee of South China University of Technology approved all animal experiments. Unless otherwise specified, the reagents were purchased from Sigma-Aldrich (St. Louis, MO, USA).

### 2.2. Neonatal Mouse Ovary Culture

After being washed in sterile phosphate-buffered saline (PBS), 3 dpp female mouse ovaries were cultured on Millipore inserts (PICMORG50, Millipore, Billerica, MA, USA) within six-well culture plates (703001, NEST, Beijing, China). The culture medium is Dulbecco’s modified Eagle’s medium/Ham’s F12 nutrient mixture, as reported previously [28]. In treatment groups, the ovaries were cultured in medium supplemented with ZnSO_4_ (0–40 μM), ISCK03 (0–5 μM; HY-101038, MedChemExpress, Monmouth Junction, NJ, USA), and/or KITL (8.06 nM; HY-P7064, MedChemExpress). ZnSO_4_ (100 mM) was prepared in ultrapure water and ISCK03 (15 mM) was prepared in dimethyl sulfoxide (DMSO) as stock solutions. The same final concentrations of DMSO (more than 0.1%) and ultrapure water were also added to the corresponding control groups. The ovaries were cultured at 37 °C with 5% CO_2_ and saturated humidity, and were collected for immunofluorescence staining, follicle counting, and gene and protein detection at the designated time points.

### 2.3. Neonatal Mouse Injection Experiment

Three-days-postpartum female mice were injected intraperitoneally three times a day with ZnSO_4_ (7.265 mg/kg, equivalent to 35 μM) and/or ISCK03 (1.777 mg/kg, equivalent to 5 μM) for two consecutive days, and each injection volume was 2–4 μL based on mouse weight of 2–4 g. The drug doses were consistent with the most effective culture concentrations, in which the mass ratio (mg/kg) replaced volume ratio (mg/L). The corresponding control mice received the same volume of saline or DMSO. At the designated time point, the ovaries were collected for follicle counting and protein detection.

### 2.4. Isolation of Oocytes from Neonatal Mice

The ovaries from mice at 3 dpp or from different cultured treatments were digested at 37 °C for 10–20 min by 0.25% trypsin, and then the digestion was terminated by adding fetal bovine serum (FBS). All oocytes were collected using glass pipettes, and the somatic cells in the medium were collected by centrifugation for RNA analysis. Most ovarian somatic cells are granulosa cells. The collected oocytes were washed and transferred to droplets of M2 medium (11320030, Thermo Fisher Scientific, Waltham, MA, USA) for subsequent use.

### 2.5. Histological and Morphological Analysis

After being fixed overnight in 4% paraformaldehyde (PFA; P1110, Solarbio, Beijing, China), the ovaries were embedded in paraffin (39601011, Leica Biosystems, Wetzlar, HE, Germany) and then were serially sectioned at a thickness of 5 µm. After being deparaffinized, hydrated, and stained with hematoxylin (H8070, Solarbio), these sections were submitted to a digital pathology scanner (VS200, Olympus Corporation, Shinjuku City, Tokyo, Japan) to capture images. The primordial follicles were counted in every fifth section, and the total primordial follicle numbers in each ovary were the average follicle numbers per section × total section numbers. The growing follicles were counted in all serial sections from one ovary. Only follicles with clearly visible oocyte nuclei were counted. All sections were counted by two independent individuals for comparison.

### 2.6. Immunofluorescent Staining

The pre-prepared ovarian sections were dewaxed and rehydrated. These sections were treated for high-temperature antigen retrieval with 0.01 M sodium citrate buffer (pH 6.0) and then were incubated in blocking 10% donkey serum for 1 h. The sections were incubated overnight with primary antibodies (Supplementary Table S1), and then were exposed to fluorescent secondary antibodies (Alexa Fluor 488- or Alexa Fluor 555-conjugated secondary antibodies, 1:200, Thermo Fisher Scientific) for 1 h at 37 °C. Finally, nuclei were stained with 4′,6-diamidino-2-phenylindole (DAPI; C1002, Beyotime, Shanghai, China) for 5–10 min.

For oocyte immunofluorescence staining, the oocytes were fixed for 30 min with 4% PFA, permeabilized for 30 min with 0.25% Triton X-100, and then blocked for 30 min with 3% bovine serum albumin (BSA). These oocytes were incubated with the Cleaved Caspase-3 primary antibody overnight at 4 °C, and then incubated with fluorescent secondary antibodies (Alexa Fluor 488- or Alexa Fluor 555-conjugated secondary antibodies) at 37 °C for 1 h. Oocyte nuclei were stained with DAPI for 5–10 min. All samples were used to capture images with a confocal microscopy (LSM 800, Carl Zeiss, Oberkochen, BW, Germany) under the same parameters. The fluorescence intensities were analyzed with ZEN (Carl Zeiss, Version 3.1). In addition, the five largest consecutive sections through the central plane of each ovary were selected for positive signal analysis. For each section, the reported fluorescence intensity value was calculated as the average of the measurements from five randomly selected follicles (five primordial or five growing follicles).

### 2.7. Zinc Measurement and ROS Staining of Oocytes

Zinc levels in oocytes were measured using the fluoZin-3 indicator dye, following the manufacturer’s instructions. The oocytes underwent staining in M2 medium at a temperature of 37 °C using 2 μM fluoZin-3 (F24195, Thermo Fisher Scientific) for 30 min, followed by a 5 min staining with Hochest33342, after which they were rinsed three times in M2 medium. Subsequently, the oocytes were transferred to glass-bottom cell culture dishes (801002, NEST) and visualized using a confocal microscopy (LSM 800, Carl Zeiss, Oberkochen, BW, Germany). For the detection of reactive oxygen species (ROS), the oocytes were treated with 10 μM DCFH-DA (S0033, Beyotime) in M2 medium at 37 °C for 30 min, and with Hochest33342 for 5 min, and then were washed three times in M2 medium. The oocytes were then placed on glass-bottom cell culture dishes and imaged using a confocal microscopy (LSM 800, Carl Zeiss, Oberkochen, BW, Germany).

### 2.8. In Situ Cell Death Detection

Apoptosis signals in ovarian sections were detected by using a Click-iT Plus TUNEL Assay (1982275; Thermo Fisher Scientific). In summary, ovarian sections that had been dewaxed and rehydrated underwent permeabilization with proteinase K at room temperature for 30 min. After two rinses with PBS, the sections were treated with a TUNEL reaction mixture for 1 h at 37 °C in darkness. The nuclei were then stained with DAPI, and the sections were examined with a confocal microscopy (LSM 800, Carl Zeiss, Oberkochen, BW, Germany).

### 2.9. Immunohistochemical Staining

Ovarian sections were stained using the rabbit IgG immunohistochemistry kit (PK-4001, Boster Biological Technology, Wuhan, China). Briefly, after deparaffinization and hydration, the ovarian sections were treated with 3% H_2_O_2_ at room temperature for a duration of 5–10 min to inhibit endogenous peroxidase activity. Subsequently, they were blocked at 37 °C for 30 min by using 5% BSA, before being incubated overnight at 4 °C with the ZIP6 primary antibody. After washing with PBS, the sections underwent incubation at 37 °C for 30 min with biotin-labeled anti-rabbit IgG, followed by another 30 min incubation with the SABC reagent at 37 °C. Immunoreactivity was visualized by using DAB substrate and counterstained by using hematoxylin. Finally, after dehydration, clearing, and mounting with neutral resin, all sections were scanned using a digital pathology scanner (VS200, Olympus Corporation, Shinjuku City, Tokyo, Japan) to capture images.

### 2.10. Western Blotting Analysis

The radio immunoprecipitation assay lysis buffer (P0013B, Beyotime), supplemented with 1 mM phenylmethylsulfonyl fluoride (ST506, Beyotime) was used to extract total protein from various ovarian samples. Then, bicinchoninic acid assay (P0012, Beyotime) was used to detect the quantity of protein. Protein samples of 15–20 μg were resuspended in SDS loading buffer (CW0044, Cwbio, Beijing, China), separated by electrophoresis on 10% or 12% SDS-polyacrylamide gels, and then transferred to pure polyvinylidene fluoride (PVDF) membranes (IPVH00010, Millipore). The membranes were blocked at room temperature for 1 h using a solution of 5% skim milk (abs9339, Absin, Shanghai, China). Next, the membranes were incubated with primary antibodies (Supplementary Table S1) overnight at 4 °C. Following tris-buffered saline tween (TBST) washes, the membranes were incubated at room temperature for 1 h with anti-mouse or anti-rabbit IgG secondary antibody (1:10,000; Zhongshan Golden Bridge Biotechnology, Beijing, China). The visualization of the protein bands on the membranes was achieved by using NcmECL Ultra Luminol/Enhancer Reagent (P10100, NCM biotech, Suzhou, China), and images were captured using the Tanon 5200 chemiluminescent imaging system (Tanon, Shanghai, China). The phosphorylated protein values were normalized to their respective total protein values, while the levels of other proteins were normalized relative to β-actin. Protein band density was quantified using ImageJ software (version 1.4.3.67; Bethesda, MD, USA). Figure S4, S5 and S7 provides uncropped scans of the Western blotting results, and Supplementary Table S3 illustrates the detailed cutting layout of the Western blot membrane.

### 2.11. RNA Isolation and Analysis

RNA extraction from 6 neonatal mouse ovaries per group was performed using ReliaPrep™ RNA Miniprep Systems (Z6111, Promega, Madison, WI, USA). Total RNA (1 µg per sample) was reverse-transcribed into cDNA using GoScript™ Reverse Transcription System (Promega, A5001). RNA extraction from 500 oocytes per group was performed using RNeasy Micro Kit (74004, Qiagen, Hilden, NW, Germany). Total RNA was reverse-transcribed into cDNA using QuantiTect Reverse Transcription Kit (205311, Qiagen). The quantitative real-time PCR (qRT-PCR) was performed with SYBR Green PCR SuperMix (AQ101, TransGen Biotech, Beijing, China) using the Light Cycler 96 system (Roche, Basel, BS, Switzerland). For the normalization of data, ribosomal protein L19 (*Rpl19*) was utilized as a control gene. The 2^−ΔΔCT^ method was applied to calculate relative mRNA levels. The details of the primers can be found in Supplementary Table S2.

### 2.12. RNA-Sequencing Analysis

Total RNA was extracted from the ovaries of control and ZnSO_4_ + ISCK03 treatment for subsequent RNA sequencing. The cDNA library was sequenced with the Illumina Novaseq6000 platform provided by Gene Denovo Biotechnology Co., Ltd. (Guangzhou, China). R and DESeq2 software were used to analyze the data. The images were accomplished by Omicstudio (www.omicstudio.cn; accessed on 29 April 2025).

### 2.13. Statistical Analysis

All experiments were conducted at least three times to ensure reliability. The biological replicates were shown by the number (*n*). Each replicate represents the mean value of data from multiple samples. Data are shown as mean ± SD. All statistical analyses were performed using GraphPad Prism (v8.3.0) and passed normality testing (Shapiro–Wilk test). Differences between two groups were assessed by two-tailed unpaired Student’s *t*-test. * *p* < 0.05, ** *p* < 0.01, *** *p* < 0.001.

## 3. Results

### 3.1. ZnSO_4_ Induces Follicular Atresia in Cultured Neonatal Mouse Ovaries in the Presence of ISCK03

**Figure 1:**
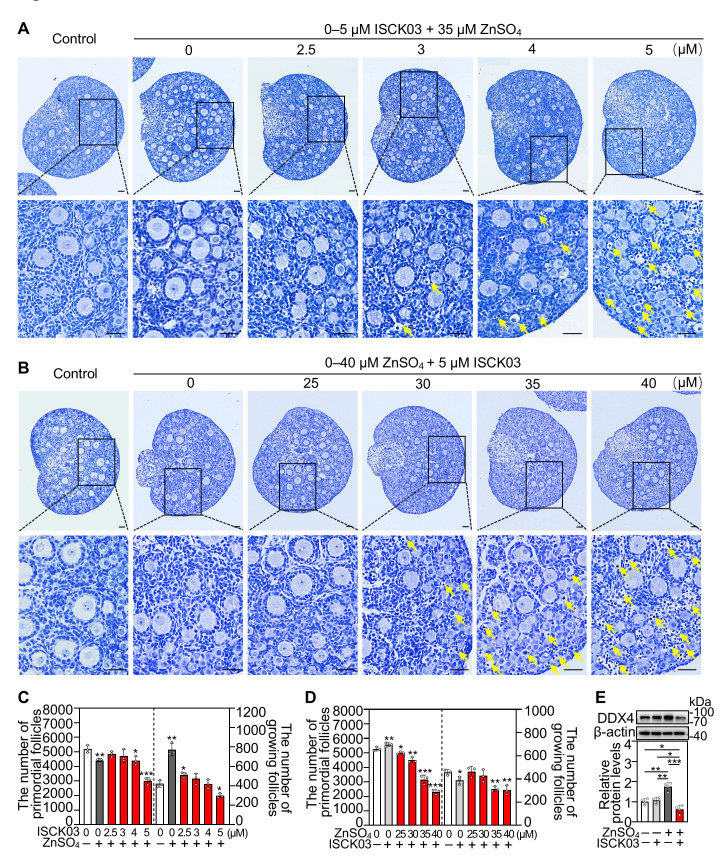
ZnSO_4_ induces follicular atresia in cultured neonatal mouse ovaries in the presence of ISCK03. Three-days-postpartum mouse ovaries were cultured without (control) or with 0–40 μM ZnSO_4_ and/or 0–5 μM ISCK03 for 48 h (**A**–**D**) or 24 h (**E**). (**A**–**D**) The comparison of ovary morphology (**A**,**B**) and primordial follicle (PF) and growing follicle (GF) numbers (**C**,**D**) in different treatments, *n* = 3, each from three ovaries. Yellow arrow represents an atretic follicle with darkly stained nucleus and vacuolated cytoplasm in the oocyte. Nuclei were stained with hematoxylin. (**E**) DDX4 protein levels in different treated ovaries, *n* = 4, each from six ovaries. The representative images are presented. Scale bars, 50 µm. Bars indicate the mean ± SD. * *p* < 0.05, ** *p* < 0.01, *** *p* < 0.001.

We cultured the neonatal mouse ovaries in a medium supplemented with ZnSO_4_ and/or ISCK03 for 48 h. The follicles were observed to survive normally in cultured neonatal mouse ovaries with ZnSO_4_ (0–55 μM) or ISCK03 (0–5 μM; Figure S1A,B) treatment alone as reported in our previous study [28]. However, ZnSO_4_ + ISCK03 treatment decreased the number of primordial and growing follicles. In the presence of 35 μM ZnSO_4_, the low concentrations of ISCK03 (<4 μM) increased the number of growing follicles (via ZnSO_4_-driven primordial follicle activation) [28], but the high concentration of ISCK03 (5 μM) significantly decreased the number of primordial and growing follicles compared with the control, accompanied by a marked increase in atretic follicles (Figure 1A,C). Conversely, in the presence of 5 μM ISCK03, the high concentrations of ZnSO_4_ (35–40 μM) significantly decreased the number of primordial and growing follicles compared with control, accompanied by a marked increase in atretic follicles (Figure 1B,D). Therefore, 35 μM ZnSO_4_ and 5 μM ISCK03 were selected for subsequent studies. Further research revealed that ZnSO_4_ + ISCK03 significantly decreased the protein levels of DEAD-box helicase 4 (DDX4, a cytoplasmic marker of oocytes) in the ovaries compared with the control (Figure 1E). These findings demonstrate that ZnSO_4_ induces follicular atresia in cultured neonatal mouse ovaries in the presence of ISCK03.

### 3.2. ZnSO_4_ Induces Zinc Overload in the Oocytes of Cultured Neonatal Mouse Ovaries in the Presence of ISCK03

**Figure 2:**
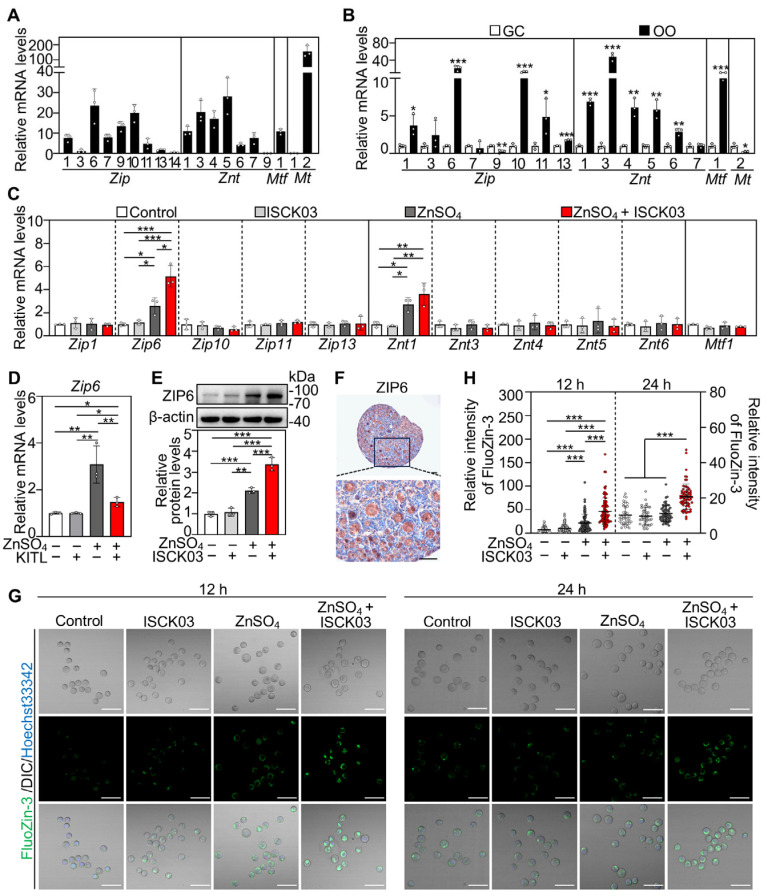
ZnSO_4_ induces zinc overload in the oocytes of cultured neonatal mouse ovaries in the presence of ISCK03. Three-days-postpartum mouse ovaries were cultured without (control) or with ZnSO_4_, ISCK03, and/or KITL for 12 h (**C**–**H**) or 24 h (**G**,**H**). (**A**) The mRNA levels of zinc homeostasis-related genes expressed in the ovaries were normalized using *Zip3* as the reference standard, *n* = 3, each from six ovaries. (**B**) The mRNA levels of zinc homeostasis-related genes in oocytes and granulosa cells, *n* = 3, each from three ovaries. OO, oocyte; GC, granulosa cell. (**C**) The mRNA levels of zinc homeostasis-related genes in different treated ovaries, *n* = 3, each from six ovaries. (**D**) The mRNA levels of *Zip6* in different treated ovaries, *n* = 3, each from six ovaries. (**E**) The protein levels of ZIP6 in different treated ovaries, *n* = 3, each from six ovaries. (**F**) Localization of ZIP6 in the ovaries (brown). (**G**,**H**) The relative fluorescence staining (green, (**G**)) and fluorescence intensity (**H**) of zinc in the oocytes under different treatments, *n* = 30–60, each from one oocyte. The representative images are presented. Scale bars, 50 µm. Bars indicate the mean ± SD. * *p* < 0.05, ** *p* < 0.01, *** *p* < 0.001.

We analyzed the expression abundance of zinc homeostasis-related genes in neonatal mouse ovaries by screening the Gene Expression Omnibus (GEO) dataset (accession: GSE232350) [28] and performing qRT-PCR. We identified zinc homeostasis-related genes that were expressed in neonatal mouse ovaries, including eight zinc import genes (*Zip1*, *Zip3*, *Zip6*, *Zip7*, *Zip9*, *Zip10*, *Zip11* and *Zip13*), six zinc export genes (*Znt1*, Z*nt3*, *Znt4*, *Znt5*, *Znt6* and *Znt7*), one zinc-responsive factor gene (*Mtf1*), and one zinc-binding gene (*Mt2*) (Figure S2A and Figure 2A). Furthermore, the mRNA levels of five zinc import genes (*Zip1*, *Zip6*, *Zip10*, *Zip11* and *Zip13*), five zinc export genes (*Znt1*, *Znt3*, *Znt4*, *Znt5* and *Znt6*), and *Mtf1* were significantly higher in oocytes than those in granulosa cells (Figure 2B).

Next, we explored the effect of ZnSO_4_ on these highly expressed genes in the oocytes. ZnSO_4_ treatment significantly increased both *Zip6* and *Znt1* mRNA levels in the ovaries compared with control, while ZnSO_4_ + ISCK03 treatment further significantly increased *Zip6* but not *Znt1* expression compared with that of ZnSO_4_ treatment (Figure 2C). Furthermore, KITL treatment reversed ZnSO_4_-induced increase in *Zip6* levels (Figure 2D), while KITL or ISCK03 treatment alone did not affect *Zip6* expression. Consistent with these, ZnSO_4_ treatment significantly increased ZIP6 protein levels, and ZnSO_4_ + ISCK03 treatment further increased ZIP6 expression in the ovaries compared with control (Figure 2E). These indicate that KITL-KIT signaling is involved in ZnSO_4_-induced ZIP6 expression. Immunohistochemical analysis revealed predominant cytoplasmic localization of ZIP6 in the oocytes within primordial and growing follicles (Figure 2F). FluoZin-3 indicator dye revealed that ZnSO_4_ treatment significantly increased zinc levels in the oocytes at the end of 12 h of culture but had no effect at the end of 24 h of culture compared with the control (Figure 2G,H). However, ZnSO_4_ + ISCK03 treatment significantly increased zinc levels in the oocytes at the end of 12 and 24 h of culture compared with the control (Figure 2G,H). These results demonstrate that ZnSO_4_ induces ZIP6 overexpression and zinc overload in the oocytes of cultured neonatal mouse ovaries in the presence of ISCK03.

### 3.3. Zinc Overload Induces Oxidative Stress and DNA Damage in the Oocytes of Cultured Neonatal Mouse Ovaries

**Figure 3:**
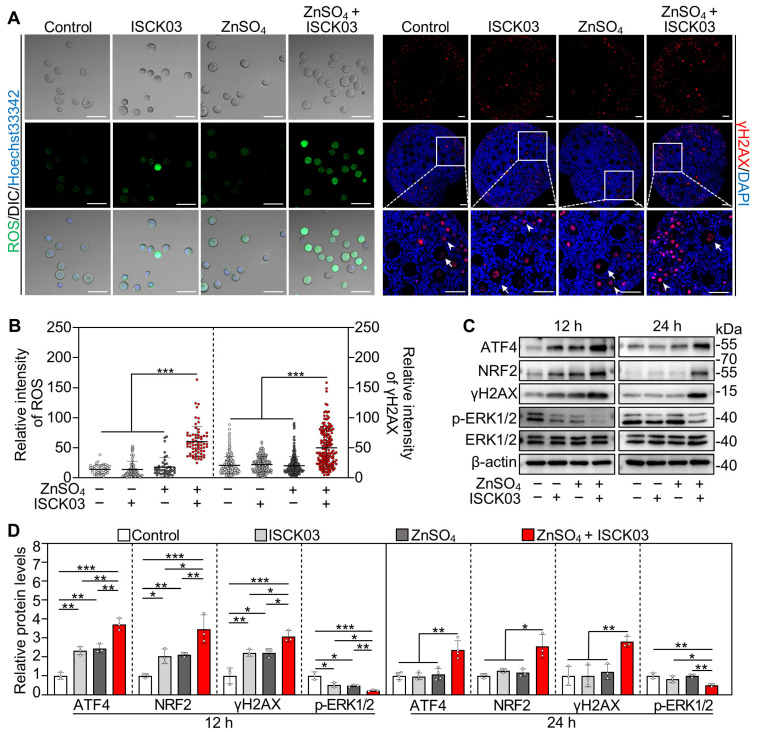
Zinc overload induces oxidative stress and DNA damage in the oocytes of cultured neonatal mouse ovaries. Three-days-postpartum mouse ovaries were cultured without (control) or with ZnSO_4_ and/or ISCK03 for 12 h (**C**,**D**) or 24 h (**A**–**D**). (**A**,**B**), The relative fluorescence staining (**A**) and fluorescence intensity (**B**) of ROS and γH2AX in the oocytes or ovaries under different treatments, *n* = 40–150, and each from one oocyte. ROS, green; γH2AX, red. Arrows, growing follicles; arrowheads, primordial follicles. (**C**,**D**) The protein levels of ATF4, NRF2, γH2AX, and p-ERK1/2 in different treated ovaries, *n* = 3 or 4, each from six ovaries. The representative images are presented. Scale bars, 50 µm. Bars indicate the mean ± SD. * *p* < 0.05, ** *p* < 0.01, *** *p* < 0.001.

We next investigated the effects of zinc overload on oxidative stress and DNA damage in the oocytes by culturing the neonatal mouse ovaries. Compared with control, ZnSO_4_ + ISCK03 treatment significantly increased the fluorescence intensity of ROS and phosphorylated H2AX (γH2AX, a DNA damage marker) in the oocytes at the end of 24 h of culture (Figure 3A,B). Consistent with this, ZnSO_4_ + ISCK03 treatment significantly increased the protein levels of activating transcription factor 4 (ATF4), nuclear factor erythroid 2-related factor 2 (NRF2) and γH2AX in the ovaries at the end of 12 and 24 h of culture compared with the control (Figure 3C,D). Furthermore, ZnSO_4_ + ISCK03 treatment significantly decreased the protein levels of phosphorylated extracellular signal-regulated kinases 1 and 2 (p-ERK1/2) in the ovaries at the end of 12 and 24 h of culture compared with the control (Figure 3C,D). However, ISCK03 or ZnSO_4_ treatment alone had no effect on the protein levels of ATF4, NRF2, γH2AX, and p-ERK1/2 in the ovaries at the end of 24 h of culture compared with the control (Figure 3C,D). Additionally, the mRNA levels of *Mt2* were significantly increased by ZnSO_4_ treatment, and were further increased by ZnSO_4_ + ISCK03 treatment in both oocytes and ovaries cultured in vitro compared with control (Figure S2B). This suggests that zinc overload results in the expression of MT to chelate excess intracellular zinc and scavenge ROS [29]. These results demonstrate that ZnSO_4_ and ISCK03 treatment-induced zinc overload results in oxidative stress and DNA damage in the oocytes of cultured neonatal mouse ovaries.

### 3.4. Zinc Overload Blocks the Autophagic Flux in the Oocytes of Cultured Neonatal Mouse Ovaries

**Figure 4:**
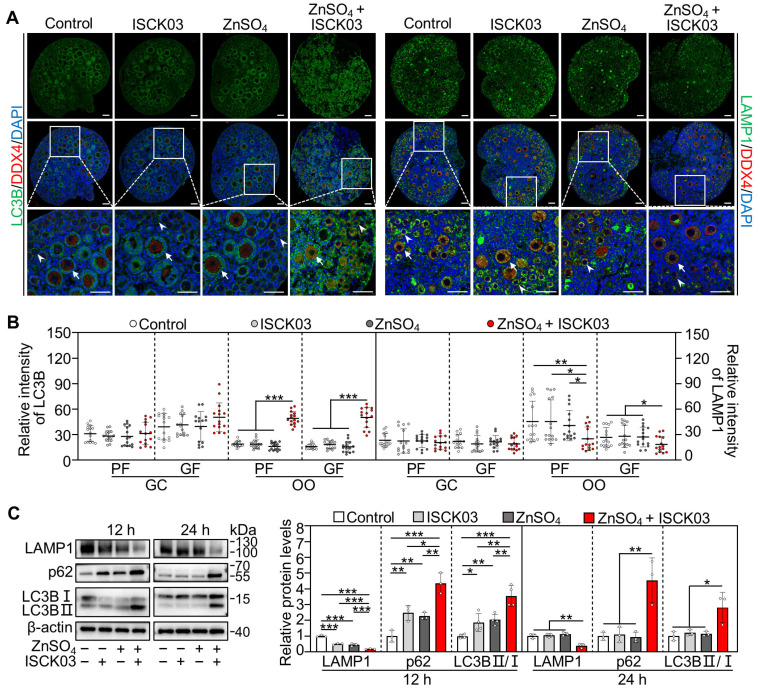
Zinc overload blocks the autophagic flux in the oocytes of cultured neonatal mouse ovaries. Three-days-postpartum mouse ovaries were cultured without (control) or with ZnSO_4_ and/or ISCK03 for 12 h (**C**) or 24 h (**A**–**C**). (**A**,**B**) The relative fluorescence staining (green, (**A**)) and fluorescence intensity (**B**) of LC3B and LAMP1 in different treated ovaries, *n* = 15, each from five follicles within one ovarian section. Arrows, growing follicles; arrowheads, primordial follicles. OO: oocyte; GC, granulosa cell. GF, growing follicle; PF, primordial follicle. (**C**) The protein levels of LAMP1, p62 and LC3B II/I in different treated ovaries, *n* = 3, each from six ovaries. The representative images are presented. Scale bars, 50 µm. Bars indicate the mean ± SD. * *p* < 0.05, ** *p* < 0.01, *** *p* < 0.001.

We further investigated the effects of zinc overload on oocyte autophagy by culturing the neonatal mouse ovaries. Compared with control, ZnSO_4_ + ISCK03 treatment significantly increased the fluorescence intensity of microtubule-associated protein 1 light chain 3 beta (LC3B) and decreased the fluorescence intensity of lysosomal-associated membrane protein 1 (LAMP1) in the oocytes at the end of 24 h of culture (Figure 4A,B). Consistent with this, ZnSO_4_ + ISCK03 treatment significantly increased the protein levels of LC3BII/I and sequestosome 1 (SQSTM1/p62), and decreased the protein levels of LAMP1 in the ovaries at the end of 12 and 24 h of culture compared with the control (Figure 4C). However, ISCK03 or ZnSO_4_ treatment alone had no effect on the protein levels of LC3BII/I, p62 and LAMP1 in the ovaries at the end of 24 h of culture compared with the control (Figure 4C). These results demonstrate that ZnSO_4_ and ISCK03 treatment-induced zinc overload blocks the autophagic flux in the oocytes of cultured neonatal mouse ovaries.

### 3.5. Zinc Overload Induces Oocyte Apoptosis in Cultured Neonatal Mouse Ovaries

**Figure 5:**
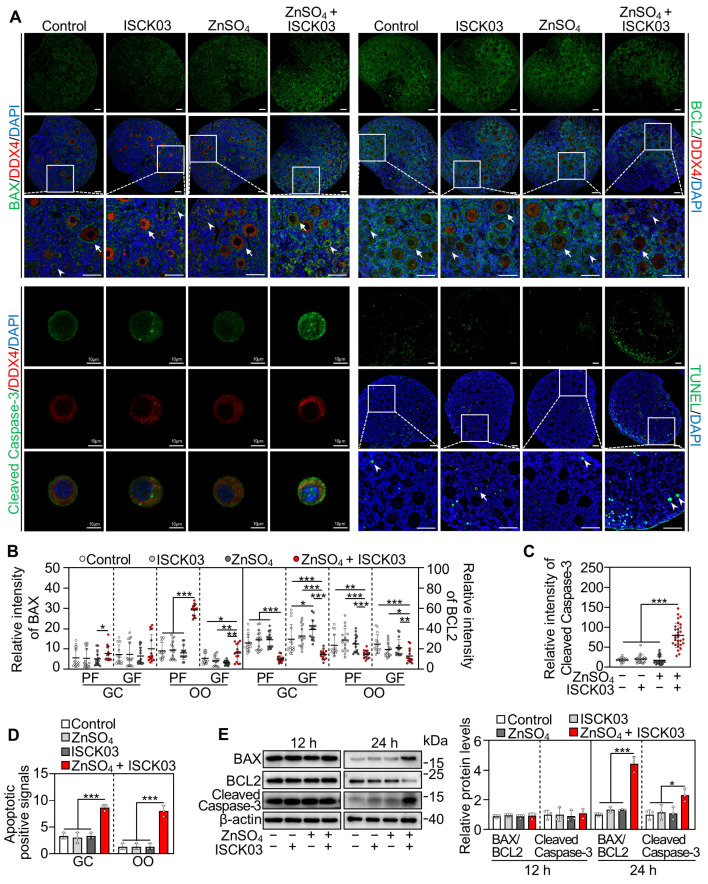
Zinc overload induces oocyte apoptosis in cultured neonatal mouse ovaries. Three-days-postpartum mouse ovaries were cultured without (control) or with ZnSO_4_ and/or ISCK03 for 24 h (**A**–**E**) or 12 h (**E**). (**A**) The relative fluorescence staining of BAX, BCL2, Cleaved Caspase-3 and TUNEL (green) in the ovaries or oocytes under different treatments. Arrows, growing follicles; arrowheads, primordial follicles. (**B**) The relative fluorescence intensity of BAX and BCL2 in different treated ovaries, *n* = 15, each from five follicles within one ovarian section. OO: oocyte; GC, granulosa cell. GF, growing follicle; PF, primordial follicle. (**C**) The relative fluorescence intensity of Cleaved Caspase-3 in the oocytes under different treatments, *n* = 30, each from one oocyte. (**D**) Quantification of TUNEL positive cells in the ovaries under different treatments, *n* = 3, each from five ovarian sections. OO: oocyte; GC, granulosa cell. (**E**) The protein levels of BAX/BCL2 and Cleaved Caspase-3 in different treated ovaries, *n* = 3, each from six ovaries. The representative images are presented. Scale bars, 50 µm or 10 µm (only Cleaved Caspase-3). Bars indicate the mean ± SD. * *p* < 0.05, ** *p* < 0.01, *** *p* < 0.001.

We also investigated the effects of zinc overload on oocyte apoptosis by culturing the neonatal mouse ovaries. Compared with the control, ZnSO_4_ + ISCK03 treatment significantly increased the fluorescence intensity of BCL2-associated X protein (BAX) and cleaved cysteine-dependent aspartate-specific protease-3 (Cleaved Caspase-3) in the oocytes, and the number of oocytes with apoptotic signals (TUNEL^+^), but decreased the fluorescence intensity of B-cell lymphoma 2 (BCL2) in the oocytes at the end of 24 h of culture (Figure 5A–D). Consistent with this, ZnSO_4_ + ISCK03 treatment significantly increased the protein levels of BAX/BCL2 and Cleaved Caspase-3 in the ovaries at the end of 24 h of culture (Figure 5E). Therefore, ZnSO_4_ and ISCK03 treatment-induced zinc overload results in oocyte apoptosis in cultured neonatal mouse ovaries.

### 3.6. Effects of Zinc Overload on the Transcriptome of Cultured Neonatal Mouse Ovaries

**Figure 6:**
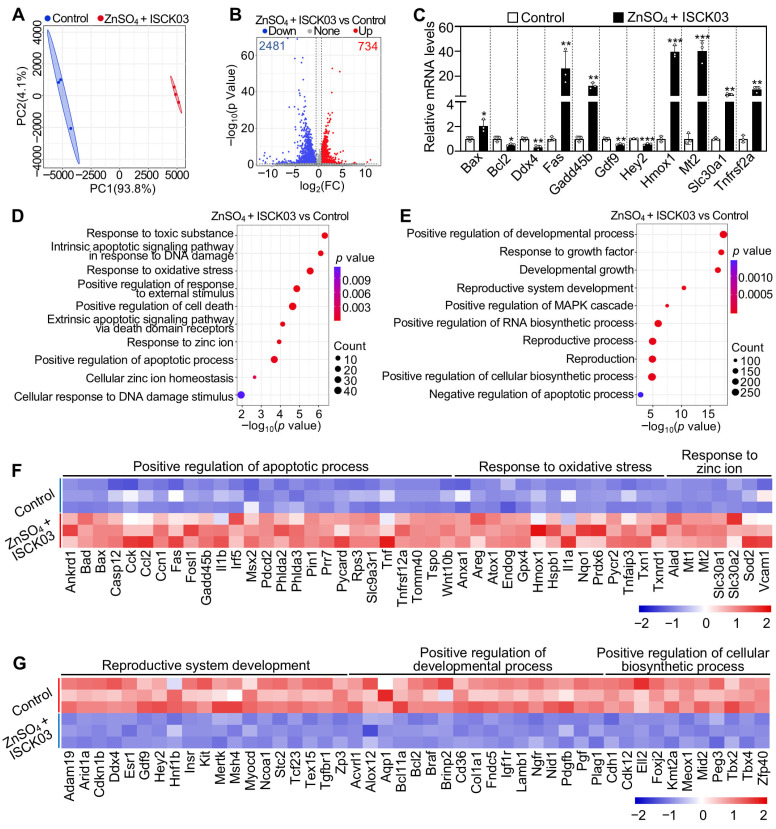
Effects of zinc overload on the transcriptome of cultured neonatal mouse ovaries. Three-days-postpartum mouse ovaries were cultured without (control) or with ZnSO_4_ and ISCK03 for 24 h. (**A**) Principal-component analysis (PCA) of samples from control and ZnSO_4_ + ISCK03 treatment groups. (**B**) Volcano plot illustrating the differentially expressed genes in ZnSO_4_ + ISCK03 treatment groups. (**C**) qRT-PCR validating changes in the representative transcripts selected from RNA-seq data, *n* = 3, each from six ovaries. (**D**,**E**) Bubble chart showing the enriched GO terms associated with the increased (**D**) and decreased (**E**) transcripts in ZnSO_4_ + ISCK03 treatment groups. (**F**,**G**) Heatmaps illustrating a group of increased (**F**) and decreased (**G**) transcripts involved in indicated biological processes between control and ZnSO_4_ + ISCK03 treatment groups. All the experiments were independently repeated three times. Bars indicate the mean ± SD. * *p* < 0.05, ** *p* < 0.01, *** *p* < 0.001.

Principal-component analysis (PCA) revealed that three biological replicates were clustered together in each group, while ZnSO_4_ + ISCK03 treatment group was clearly separated from the control group (Figure 6A). ZnSO_4_ + ISCK03 treatment induced changes in a total of 3215 transcripts (including 734 upregulated and 2481 downregulated transcripts) compared with the control (Figure 6B). The changes in the expression of representative transcripts were verified through qRT-PCR (Figure 6C). Gene enrichment analysis revealed that the upregulated transcripts in ZnSO_4_ + ISCK03 treatment were associated with DNA damage, oxidative stress, cell death, apoptotic processes, zinc ion homeostasis, and response to zinc ion (Figure 6D,F). Concurrently, the downregulated transcripts in ZnSO_4_ + ISCK03 treatment were associated with developmental processes, the response of growth factor, reproductive system development, MAPK cascade, and RNA biosynthetic processes (Figure 6E,G). Therefore, ZnSO_4_ and ISCK03 treatment-induced zinc overload results in the upregulation of genes associated with oxidative stress, DNA damage, and apoptosis in cultured neonatal mouse ovaries.

### 3.7. ZnSO_4_ Induces Mouse Oocyte Apoptosis in the Presence of ISCK03 In Vivo

**Figure 7:**
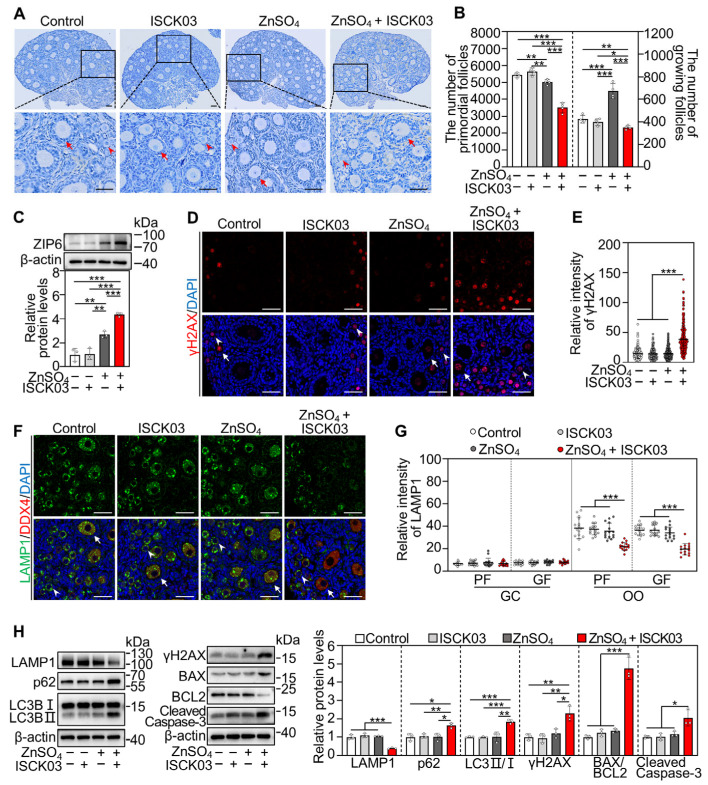
ZnSO_4_ induces mouse oocyte apoptosis in the presence of ISCK03 *in vivo*. Three-days-postpartum female mice were intraperitoneally injected with 7.265 mg/kg ZnSO_4_ and/or 1.777 mg/kg ISCK03 three times a day for two consecutive days. The ovaries were collected 48 h (**A**,**B**), 12 h (**C**), or 24 h (**D**–**H**) after the end of injection. (**A**,**B**) A comparison of ovary morphology (**A**) and primordial follicle (PF) and growing follicle (GF) number (**B**), *n* = 4, each from three ovaries. Nuclei were stained by hematoxylin. Arrows, growing follicles; arrowheads, primordial follicles. (**C**) ZIP6 protein levels in different treated ovaries, *n* = 3, each from six ovaries. (**D**,**E**) The relative fluorescence staining (**D**) and fluorescence intensity (**E**) of γH2AX (red) in different treated ovaries, *n* = 100–150, and each from one oocyte. Arrows, growing follicles; arrowheads, primordial follicles. (**F**,**G**) The relative fluorescence staining (**F**) and fluorescence intensity (**G**) of LAMP1 (green) in different treated ovaries, *n* = 15, each from five follicles within one ovarian section. Arrows, growing follicles; arrowheads, primordial follicles. OO: oocyte; GC, granulosa cell. GF, growing follicle; PF, primordial follicle. (**H**) The protein levels of LAMP1, p62, LC3BII/I, BAX, BCL2 and Cleaved Caspase-3 in different treated ovaries, *n* = 3, each from six ovaries. The representative images are presented. Scale bars, 50 µm. Bars indicate the mean ± SD. * *p* < 0.05, ** *p* < 0.01, and *** *p* < 0.001.

Female mice at 3 dpp were injected intraperitoneally three times a day with ZnSO_4_ and/or ISCK03 for two consecutive days. Compared with the control, ZnSO_4_ + ISCK03 treatment significantly decreased the number of primordial and growing follicles (Figure 7A,B). ZnSO_4_ treatment significantly increased ZIP6 protein levels, and ZnSO_4_ + ISCK03 treatment further increased ZIP6 protein levels in the ovaries compared with the control (Figure 7C). Additionally, ZnSO_4_ + ISCK03 treatment significantly increased the fluorescence intensity of γH2AX in the oocytes and the protein levels of γH2AX, LC3BII/I, p62, BAX/BCL2 and Cleaved Caspase-3 in the ovaries compared with the control (Figure 7D,E,H, and Figure S3A). Meanwhile, ZnSO_4_ + ISCK03 treatment significantly decreased LAMP1 fluorescence intensity in the oocytes and its protein levels in the ovaries compared with the control (Figure 7F,G,H, and Figure S3B). Therefore, ZnSO_4_ and ISCK03 treatment-induced zinc overload increases ZIP6 expression, DNA damage, autophagic flux blockade, and apoptosis of oocytes in neonatal mice.

## 4. Discussion

**Figure 8:**
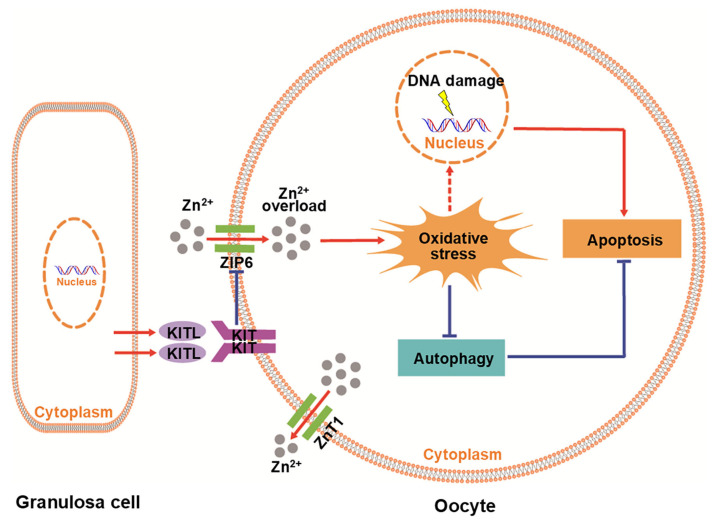
Mechanism of the KITL-KIT signaling in maintaining oocyte zinc homeostasis. KITL secreted by granulosa cells binds to KIT on oocytes to inhibit zinc-induced overexpression of ZIP6, and then prevents zinc overload and oocyte apoptosis.

KITL-KIT signaling is crucial for primordial follicle formation and activation and follicular development (Figure S6). In our study, ZnSO_4_ caused zinc overload in the oocytes of neonatal mouse ovaries in the presence of the KITL-KIT signaling inhibitor ISCK03 through the overexpression of ZIP6, ultimately resulting in the apoptosis of oocytes within primordial and growing follicles (Figure 8). Together, KITL-KIT signaling is involved in maintaining zinc homeostasis in the oocytes of neonatal mouse ovaries.

We found that the mRNA levels of zinc importer genes *Zip1*, *Zip6*, *Zip10*, *Zip11,* and *Zip13* in the oocytes within primordial and growing follicles were higher than those in granulosa cells, and *Zip6* displayed the highest expression abundance in the oocytes within primordial and growing follicles. These are consistent with the previous study showing that the mRNA levels of *Zip6* in the fully grown oocytes are significantly higher than those in granulosa cells [30]. ZIP6 is typically located in the plasma membrane of fully grown oocytes [21], but is also mainly expressed in the cytoplasm of oocytes within primordial and growing follicles [31]. Consistent with this, ZIP6 staining was observed primarily in the cytoplasm of oocyte within primordial and growing follicles in our study. ZIP6, an integral membrane protein, is possibly located in the vesicles of oocyte cytoplasm and involved in zinc transport.

Twenty-four hours of ZnSO_4_ treatment had no effect on DNA damage or cell apoptosis. In our recent study, oral administration with the same concentration of ZnSO_4_ for one week significantly increased oocyte quantity and ameliorated fertility deficits in aged mice [28]. These results indicate that ZnSO_4_ treatment has no obvious negative impact on the subsequent health of follicles. Sulfate ions (SO_4_^2−^) exhibit high chemical inertness in physiological environments and rarely participate in biological reactions. Thus, ZnSO_4_, as the source of zinc, is usually used to improve erythrocyte deformability and aggregation in beta-thalassemia patients, and to reduce zinc deficiency-induced neuronal apoptosis [32,33]. In our study, ZnSO_4_ increased the mRNA and protein levels of ZIP6, accompanied by the increase in zinc levels in the oocytes of neonatal mouse ovaries. It has been reported that ZIP6-promoted zinc influx participates in oocyte meiotic maturation [21]. Therefore, the elevated extracellular zinc promotes ZIP6 expression and then drives zinc influx into the oocytes of neonatal mouse ovaries.

Zinc further increased the mRNA and protein levels of ZIP6 in the presence of ISCK03, accompanied by zinc overload in the oocytes of neonatal mouse ovaries. Thus, zinc results in zinc overload in the oocytes of neonatal mouse ovaries by inducing ZIP6 overexpression when KITL-KIT signaling is inhibited by ISCK03. A previous report has shown that ZIP6-mediated zinc influx participates in oocyte meiotic maturation [21]. This may be due to the impairment of the KITL-KIT signaling caused by the disruption of cellular connections between granulosa cells and oocytes, resulting in an increase in ZIP6 expression. It has been reported that the inhibition of KITL-KIT signaling enhances neuronal sensitivity to glutamate [10,14]. In the present study, the inhibition of KITL-KIT signaling may enhance the sensitivity of ZIP6 expression to zinc in the oocytes of neonatal mouse ovaries.

*Zip6* is located on chromosome 18 (18 E1), while *Znt1* resides on chromosome 1 (1 H3) in the mouse genome, suggesting there is no direct genetic correlation between the two genes. ZnSO_4_ and ZnSO_4_ + ISCK03 treatments increased the expression of *Zip6* in the oocytes within primordial and growing follicles, resulting in an increase in zinc in the oocytes of neonatal mouse ovaries. ZnT1 is the only zinc transporter predominantly localized to the plasma membrane in mammalian cells [34]. The upregulation of *Znt1* mRNA levels observed in the present study may be attributed to a negative feedback response of zinc overload. In neonatal mouse ovary, *Mt2* was highly expressed, *Mt1* was slightly expressed, and *Mt3* and *Mt4* were barely expressed (Figure 2A and Figure S2A). ZnSO_4_ + ISCK03 treatment increased the expression of both *Mt1* and *Mt2* (Figure 6F), suggesting MT1 and MT2 are involved in zinc buffering in oocytes.

We also found that zinc overload caused oxidative stress, DNA damage, and ERK1/2 dephosphorylation in the oocytes of neonatal mouse ovaries. This is consistent with previous reports that zinc overload activates oxidative stress by upregulating heme oxygenase 1 (HMOX1), and then induces DNA damage in HeLa S3 cells and ERK1/2 dephosphorylation via ataxia telangiectasia mutated (ATM)-independent mechanisms in various cells [35,36]. The severe DNA damage triggers apoptosis in various cells [37]. Therefore, we speculate that zinc overload results in DNA damage through oxidative stress, ultimately triggering oocyte apoptosis in neonatal mouse ovaries.

Oxidative stress has been shown to induce autophagy to prevent the apoptosis of bovine mammary epithelial cells [38]. In our study, zinc overload induced autophagic flux blockade, accompanied by an increase in NRF2 protein levels. These results are consistent with previous studies showing that zinc overload induces cardiomyocyte damage through excessive oxidative stress-mediated mitophagy impairment [39,40]. Autophagic flux blockade is often accompanied by the activation of the NRF2 antioxidant pathway [41]. Therefore, autophagic flux blockade is also involved in oocyte apoptosis in neonatal mouse ovaries. Together, we conclude that zinc overload results in oocyte apoptosis in neonatal mouse ovaries via oxidative stress-driven dual mechanisms: irreversible DNA damage in the nucleus and autophagic flux blockade in the cytoplasm of oocytes. In the present study, ZnSO_4_ + ISCK03 induced zinc overload, oxidative stress, and autophagic flux blockade (Figure 2, Figure 3 and Figure 4). Furthermore, the intraperitoneal injection of a high concentration of ZnSO_4_ (28.754 mg/kg, equivalent to 100 μM) in neonatal mice resulted in extensive apoptosis of primordial and growing follicles (Figure S9). These are consistent with the previous studies that zinc overload triggers oocyte and cardiomyocyte apoptosis through oxidative stress [27,40]. Thus, ZnSO_4_ + ISCK03 induces oxidative stress and autophagic flux blockage via zinc overload.

ZnSO_4_ + ISCK03 could lead to zinc overload in oocytes by upregulating ZIP6 expression. However, a specific ZIP6 inhibitor for further study is lacking. Thus, we cannot rule out other factor(s) that also participate in ZnSO_4_ + ISCK03-induced zinc overload. The experiments were conducted using neonatal mice. The effect of ZnSO_4_ + ISCK03 on ovarian follicles in pubertal and adult mice remains to be investigated. RNA-seq (GSE107746) data analysis revealed that *KIT* and *ZIP6* are highly expressed in the oocytes of human primordial follicles (Figure S8), whether ZnSO_4_ + ISCK03 induces zinc overload in human oocytes requires further investigation. All these will be beneficial to the clinical diagnosis and treatment of zinc overload.

In conclusion, our study indicates that granulosa cell-secreted KITL is involved in maintaining zinc homeostasis in the oocytes of neonatal mouse ovaries. This finding not only reveals a novel function of granulosa cells in supporting oocyte homeostasis, but also provides a theoretical basis for identifying individuals susceptible to zinc dyshomeostasis caused by impaired KITL-KIT signaling.

## Data Availability

The original data presented in the study are openly available in the Gene Expression Omnibus (GEO) repository under accession number GSE308150. The remaining data supporting the findings of this study can be directed to the corresponding authors.

## Supplementary Materials

The following supporting information can be downloaded at: https://www.mdpi.com/article/10.3390/antiox14111345/s1, Figure S1: Effect of ISCK03 on primordial and growing follicle number in cultured neonatal mouse ovaries; Figure S2: Expression of zinc homeostasis-related genes in cultured neonatal mouse ovaries (GSE232350); Figure S3: Effect of ZnSO_4_ and ISCK03 on follicles in mice in vivo; Figure S4: Uncropped scans of the Western blotting results in Figure 1, Figure 2, Figure 3, Figure 4 and Figure 5; Figure S5: Uncropped scans of the Western blotting results in Figure 7; Figure S6: Single-cell RNA sequencing (scRNA-seq) analysis reveals *Kitl* and *Kit* expression in neonatal mouse ovaries (GSE263836); Figure S7: Representative composite of trimmed Western blot membranes from Figure 1, Figure 3, Figure 4 and Figure 5; Figure S8: Expression of *KITLG*, *KIT*, and *ZIPs* in human primordial follicles (GSE107746); Figure S9: Effect of high-concentration ZnSO_4_ on primordial and growing follicles in neonatal mice in vivo; Table S1: List of primary antibodies used in immune detection in this study; Table S2: Primers for qRT-PCR used in this study; Table S3: Detailed time for the Western blot membrane trimming workflow. Western Blot Data Integrity Statement.

## Abbreviations

The following abbreviations are used in this manuscript:

Akt	protein kinase B
ATF4	activating transcription factor 4
ATM	ataxia telangiectasia mutated
BAX	BCL2-associated X protein
BCL2	B-cell lymphoma 2
BSA	bovine serum albumin
Caspase-3	cysteine-dependent aspartate-specific protease-3
DAPI	4′,6-diamidino-2-phenylindole
DDX4	DEAD-box helicase 4
DMEM/F12	Dulbecco’s modified Eagle’s medium/Ham’s F12 nutrient mixture
DMSO	dimethyl sulfoxide
dpp	days postpartum
FBS	fetal bovine serum
GAPDH	glyceraldehyde-3-phosphate dehydrogenase
GC	granulosa cell
GEO	Gene Expression Omnibus
GF	growing follicle
γH2AX	phosphorylated H2AX
HMOX1	heme oxygenase 1
JAK	Janus kinase
KIT	proto-oncogenic receptor tyrosine kinase
KITL	KIT ligand
LAMP1	lysosomal-associated membrane protein 1
LC3B	microtubule-associated protein 1 light chain 3 beta
MAPK	mitogen-activated protein kinase
mTOR	mammalian target of rapamycin
MTF1	metal regulatory transcription factor 1
MTs	metallothioneins
NRF2	nuclear factor erythroid 2-related factor 2
OO	oocyte
PBS	phosphate-buffered saline
PCA	principal-component analysis
PF	primordial follicle
PFA	paraformaldehyde
PI3K	phosphoinositide 3-kinase
p-ERK1/2	phosphorylated extracellular signal-regulated kinases 1 and 2
PVDF	polyvinylidene fluoride
qRT-PCR	quantitative real-time PCR
ROS	reactive oxygen species
SDS	sodium dodecyl sulfate
SMAD	Sma- and Mad-related protein
p62/SQSTM1	sequestosome 1
ZIPs	Zrt/Irt-like proteins
ZnTs	zinc transporters
