# Supplementary material for: Granulosa Cell-Secreted KITL Is Involved in Maintaining Zinc Homeostasis in the Oocytes of Neonatal Mouse Ovaries
Source: Antioxidants (Basel). 2025 Nov 10;14(11):1345. doi: 10.3390/antiox14111345 (PMC12649686; doi:10.3390/antiox14111345)
Supplement: Supplementary file 1 [file antioxidants-14-01345-s001.zip › antioxidants-3934256-Supplement materials-2nd proof-aus claimed no change after 2nd proof done.pdf]

Supporting information

Granulosa cell-secreted KITL is involved in maintaining zinc homeostasis in the oocytes of neonatal mouse ovaries

Yan Du[1, 2], Lincheng Han[1], Hongwei Wei[1], Xiaodan Zhang[1], Wenbo Zhang[1], Yashuang Weng[1], Weiyong Wang[1], Luchun Zhang[1], Sihui He[1], Meijia Zhang[1*], Jingjie Li[2*]

[1]The Innovation Centre of Ministry of Education for Development and Diseases, School of Medicine, South China University of Technology, Guangzhou 510006, Guangdong, China.

[2]Reproductive Medicine Center, The Sixth Affiliated Hospital, Sun Yat-sen University, Guangzhou 510080, Guangdong, China.

[*]Corresponding author: Jingjie Li (13580425302, lijjie3@mail.sysu.edu.cn); Meijia Zhang (13581782404, zhangmeijia@scut.edu.cn)

Supplementary Figures

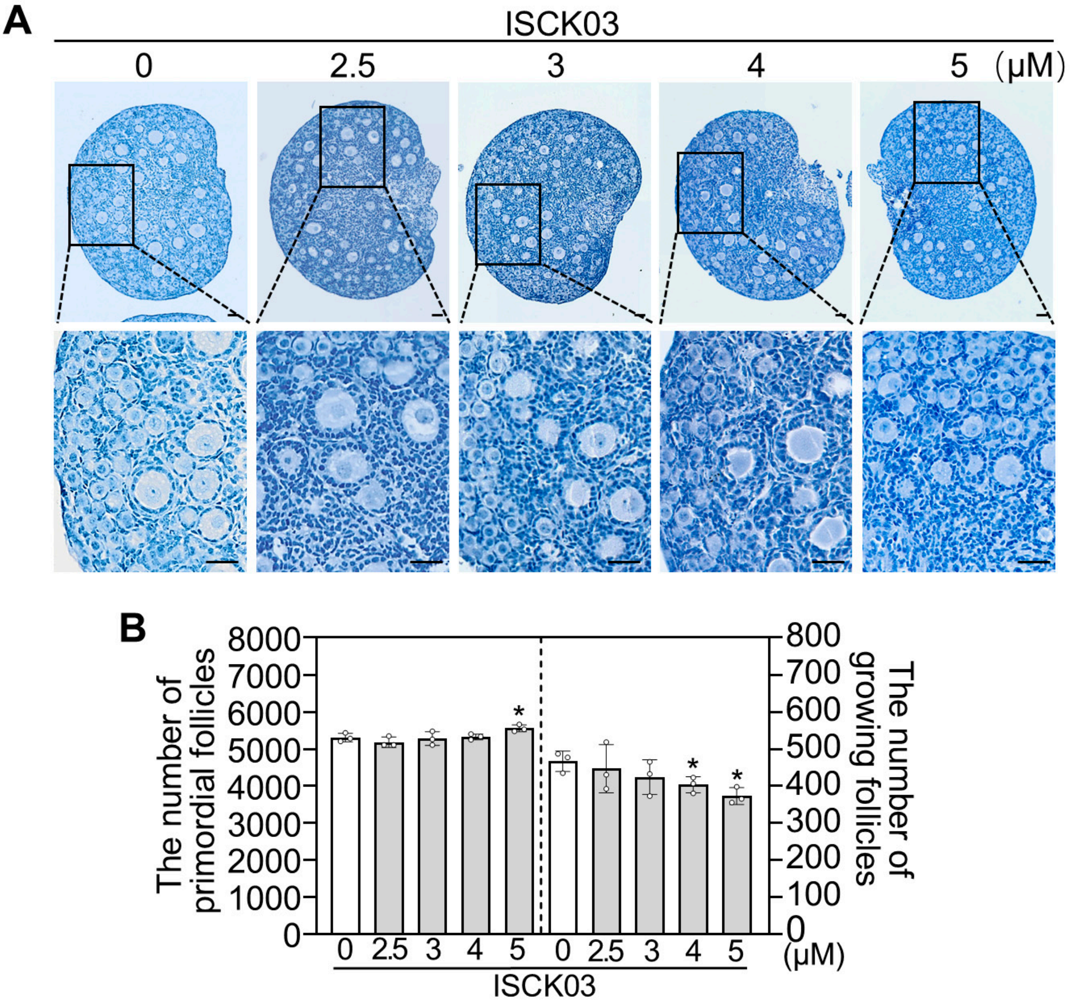

Figure S1. Effect of ISCK03 on primordial and growing follicle number in cultured neonatal mouse ovaries. 3 dpp mouse ovaries were cultured without (control) or with 0-5 μM ISCK03 for 48 hours (A-B). The comparison of ovary morphology (A) and primordial follicle (PF) and growing follicle number (GF. B) in different treatments, n = 3, and each from 3 ovaries. Nuclei were stained with hematoxylin. The representative images are presented. Scale bars, 50 μm. Bars indicate the mean ± SD. *$p < 0.05$.

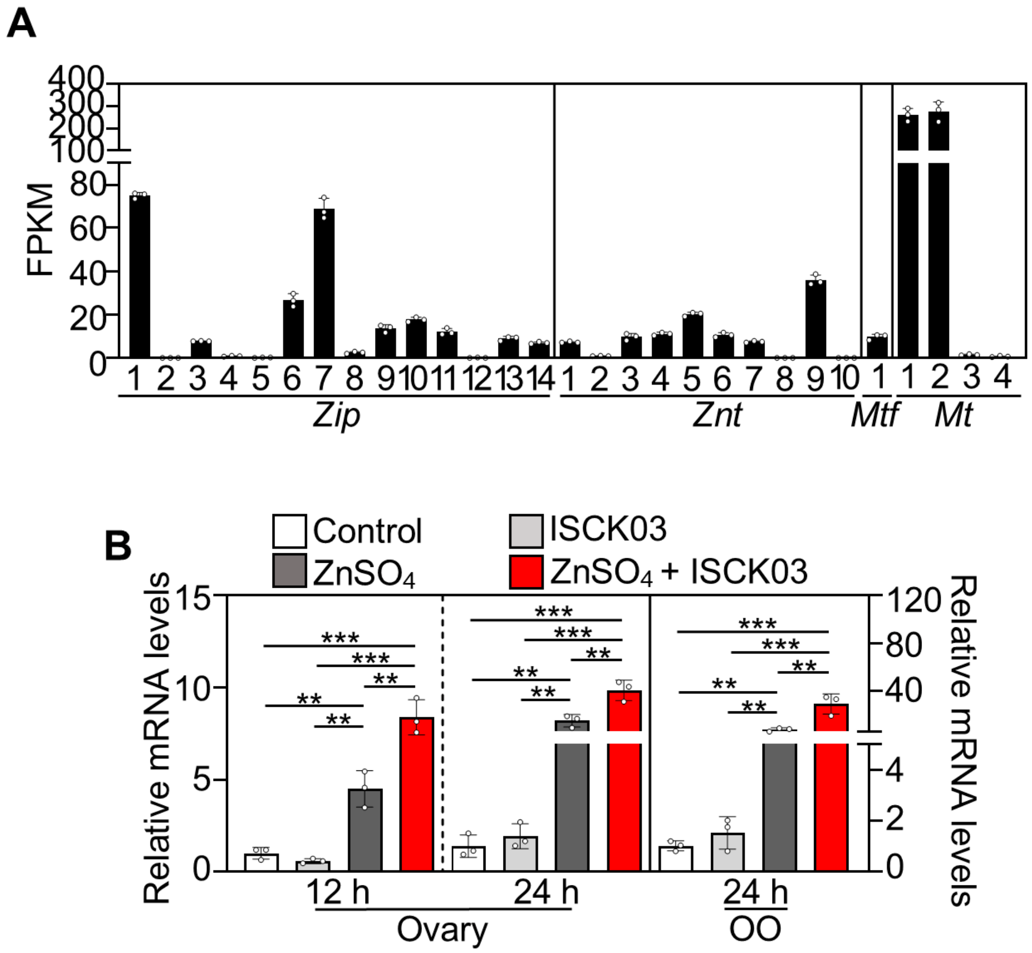

Figure S2. Expression of zinc homeostasis-related genes in cultured neonatal mouse ovaries. Analysis of zinc homeostasis-related gene expression in neonatal mouse ovaries, the values were from published data (GSE232350). **A**, Fragments Per Kilobase of exon model per Million mapped fragments (FPKM) values of zinc homeostasis-related genes. **B**, 3 dpp mouse ovaries were cultured without (control) or with $ZnSO_4$ and/or ISCK03 for 12 hours. The mRNA levels of *Mt2* in the ovaries and oocytes under different treatments are shown. OO, oocyte. Left Y-axis: 12-hour ovarian cultures; Right Y-axis: 24-hour ovaries and oocytes. Bars indicate the mean ± SD. $^{**}p < 0.01$, $^{***}p < 0.001$.

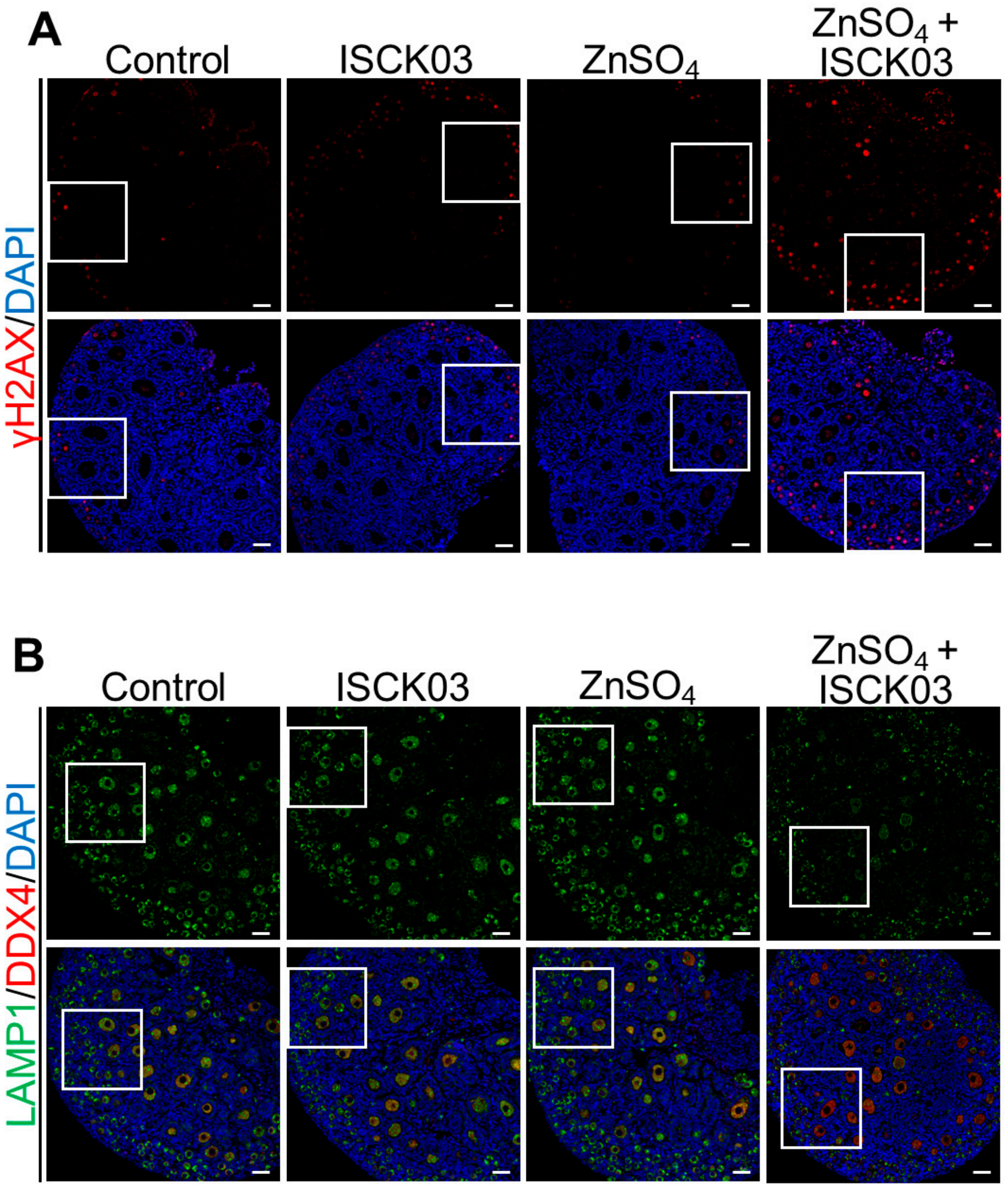

Figure S3. Effect of $ZnSO_4$ and ISCK03 on follicles in mice *in vivo*. Female mice at 3 dpp were intraperitoneally injected with 7.265 mg/kg $ZnSO_4$ and/or 1.777 mg/kg ISCK03 three times a day for two consecutive days. The ovaries were collected after 12 hours of the end of injection. **A**, The relative fluorescence staining of γH2AX (red) in different treated ovaries. **B**, The relative fluorescence staining of LAMP1 (green) in different treated ovaries. The amplified views of the boxed area are shown in Figure 7D and F. The representative images are presented. Scale bars, 50 μm.

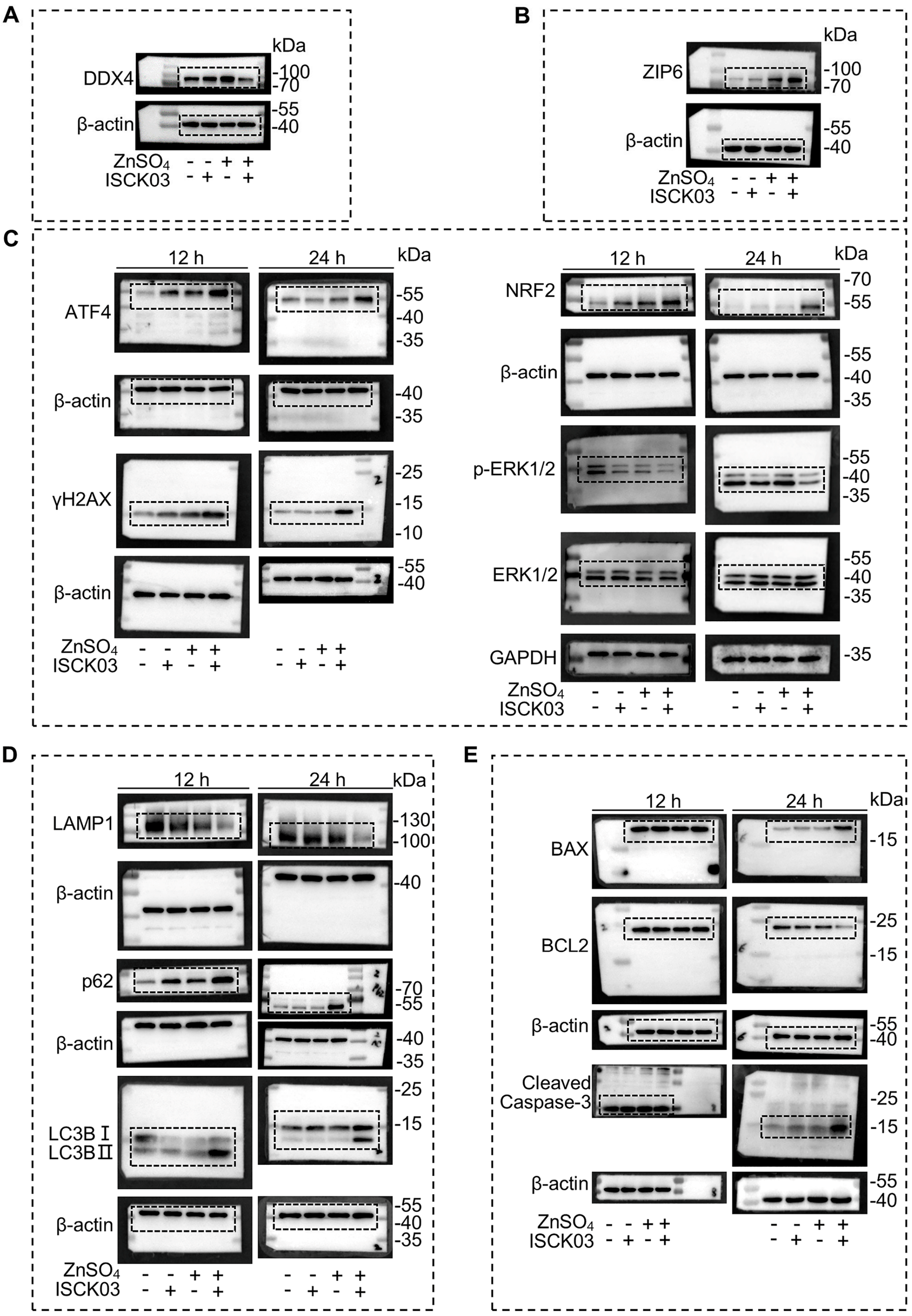

Figure S4. Uncropped scans of the Western blotting results in Figure 1, 2, 3,4 and 5. The blots in the black dashed line boxes were used in Figure 1E (**A**), 2E (**B**), 3C (**C**), 4C (**D**) and 5E(**E**).

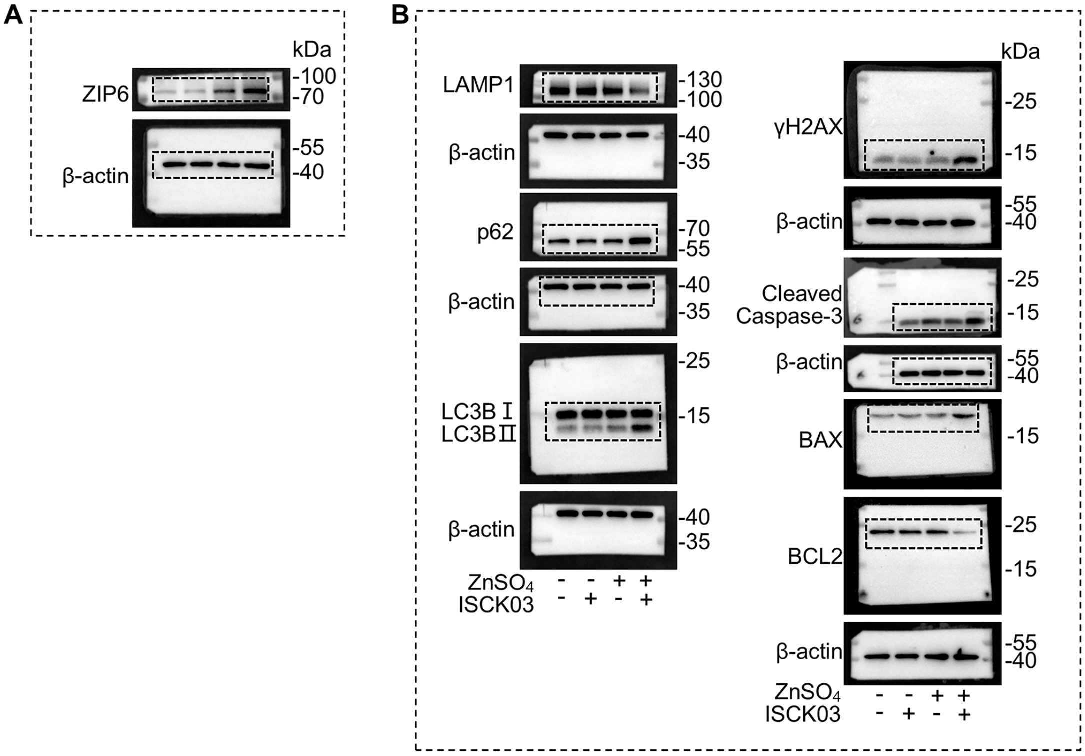

Figure S5. Uncropped scans of the Western blotting results in Figure 7. The blots in the black dashed line boxes were used in Figure 7C(**A**) and H(**B**).

.

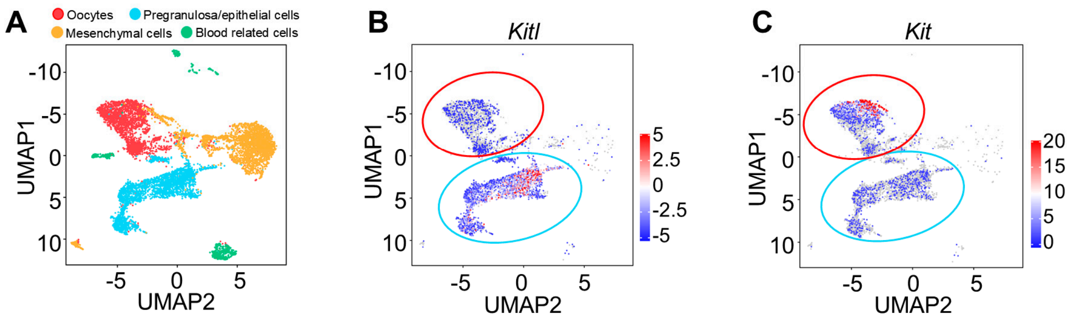

Figure S6. Single-cell RNA sequencing (scRNA-seq) analysis reveals *Kitl* and *Kit* expression in neonatal mouse ovaries. **A**, uMAP plots of ovarian cells based on four cell types (oocytes, pregranulosa cells, mesenchymal cells and blood-related cells). **B**. uMAP visualization showing the expression distribution of *Kitl* in pregranulosa cell and oocyte populations. **C**. uMAP visualization showing the expression distribution of *Kit* in pregranulosa cell and oocyte populations. The areas circled in red indicate the oocyte populations, while those circled in blue indicate the pregranulosa cell populations. The values were from published data (GSE263836).

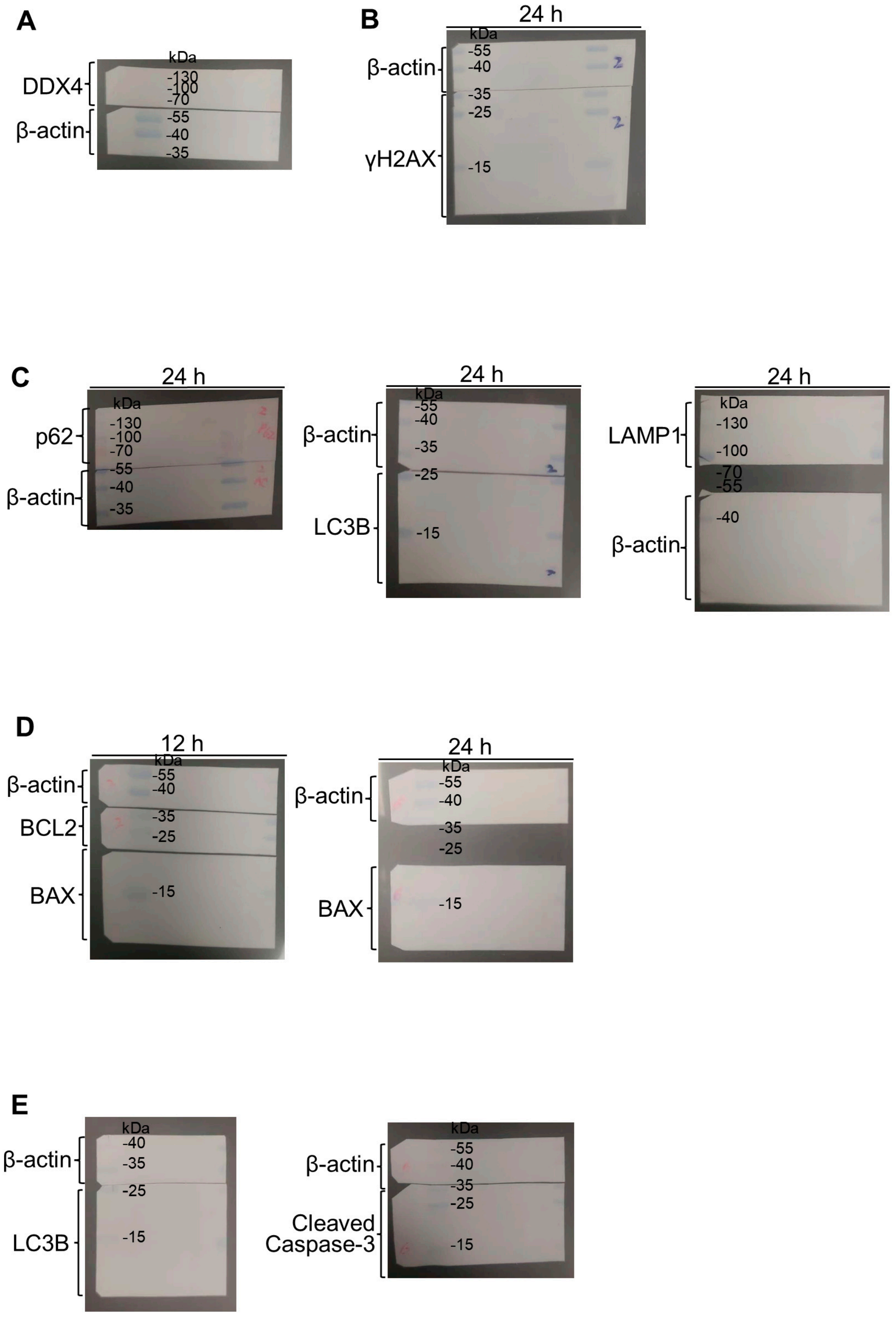

Figure S7. Representative composite of trimmed Western blot membranes**.** The blots were used in Figure 1E (**A**), ,3C (**B**), 4C (**C**), 5E(**D**) and 7H(**E**). All membrane trimming procedures were performed at the School of Medicine, South China University of Technology, and all blots were exposed using the Tanon 5200 chemiluminescent imaging system (Tanon, Shanghai, China). All other Western blotting bands in the present study were obtained using the trimmed intact membranes.

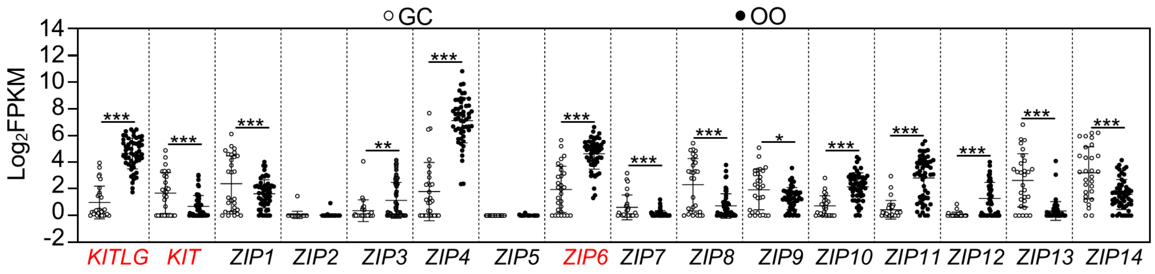

Figure S8. Expression of *KITLG*, *KIT*, and *ZIPs* in human primordial follicles. The expression levels of *KITLG*, *KIT* and *ZIP1-14* in granulosa cells (n = 8 follicles) and oocytes (n = 17 follicles) of human primordial follicles (GSE107746). GC, granulosa cell; OO, oocyte. Bars indicate the mean ± SD. *$p < 0.05$, **$p < 0.01$, ***$p < 0.001$.

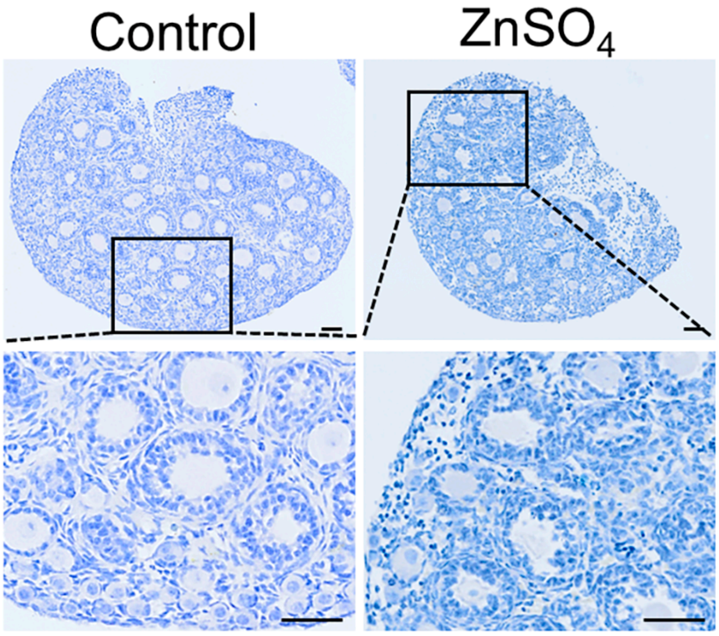

Figure S9. Effect of high-concentration $ZnSO_4$ on primordial and growing follicles in neonatal mice ***in vivo*.** 3 dpp female mice were intraperitoneally injected with 28.754 mg/kg $ZnSO_4$ or normal saline three times a day for two consecutive days. The ovaries were collected after 48 hours at the end of injection. The corresponding representative images are presented. Scale bars, 50 μm.

Supplementary Tables

Table S1. List of primary antibodies used in immune detection in this study.

Antibody	Catalog code	Source	Host	Dilution	
				**IF/IHC**	**WB**
ATF4	11815	CST	Rabbit	—	1:1000
BAX	50599-2-Ig	Proteintech	Rabbit	1:200	1:1500
BCL2	26593-1-AP	Proteintech	Rabbit	1:200	1:1500
Cleaved Caspase-3	9664	CST	Rabbit	1:100	1:500
DDX4	ab27591	Abcam	Mouse	1:200	1:1000
ERK1/2	4370	CST	Rabbit	—	1:1000
p-ERK1/2	AP0234	ABclonal	Rabbit	—	1:1000
GAPDH	2118	CST	Rabbit	—	1:1000
LAMP1	ab320851	Abcam	Rabbit	1:200	1:1000
LC3B	NB100-2220	Novus Biologicals	Rabbit	1:200	1:1000
NRF2	sc-365949	Santa Cruz	Mouse	—	1:500
p62	PTM-5483	Novus Biologicals	Rabbit	—	1:1000
ZIP6	14236-1-AP	Proteintech	Rabbit	1:200	1:1000
β-actin	4967	CST	Rabbit	—	1:1000
γH2AX	ab22551	Abcam	Mouse	—	1:1000
γH2AX	ab206900	Abcam	—	1:300	—

IF: Immunofluorescence; IHC: Immunohistochemistry; WB: Western blotting

Table S2. Primers for qRT–PCR used in this study.

Genes	Forward primers (5’-3’)	Reverse primers (5’-3’)
Bax	TTTCATCCAGGATCGAGCAGG	GCAAAGTAGAAGAGGGCAACCAC
Bcl2	CTACCGTCGTGACTTCGCA	TACCCAGCCTCCGTTATCC
Ddx4	GCTTCATCAGATATTGGCGAGT	GCTTGGAAAACCCTCTGCTT
Fas	TATCAAGGAGGCCCATTTTGC	TGTTTCCACTTCTAAACCATGCT
Gadd45b	CAACGCGGTTCAGAAGATGC	GGTCCACATTCATCAGTTTGGC
Gdf9	TCTTAGTAGCCTTAGCTCTCAGG	TGTCAGTCCCATCTACAGGCA
Hey2	CAAGGATCTGCCAAGTTAGAAAAG	TGTCAAGCACTCTCGGAATC
Hmox1	AAGCCGAGAATGCTGAGTTCA	GCCGTGTAGATATGGTACAAGGA
Rpl19	CTGAAGGTCAAAGGGAATGTGTTC	TGGTCAGCCAGGAGCTTCTTG
Tnfrsf12a	GTGTTGGGATTCGGCTTGGT	GTCCATGCACTTGTCGAGGTC
Mtf1	ACACCTTCGTCTGTAATCAGGA	CTGCACGTCACACTCAAATGG
Mt1	AAGAGTGAGTTGGGACACCTT	CGAGACAATACAATGGCCTCC
Mt2	GCCTGCAAATGCAAACAATGC	AGCTGCACTTGTCGGAAGC
Zip1	AGGTCAGGTGCTAACCATGAA	CTGTTCCTTGTAAGCCAGCGT
Zip3	CCATGGTTCACACACAGAGG	AGGGTCCCTGAGGTCACTTT
Zip6	TTCCTGTCTCTGCTGGGAGT	TGTGCTGATGACTTGCATGA
Zip7	TGAAAGCATCTGGCATGGG	TGGAGGCTATCGTGGGAGTG
Zip9	GCATTAGAGGCAGCAGGAAC	GCATTAAGGCATCCACACCT
Zip10	TACCCACCAGCATTTTCACA	TCACTGTGAGCAACGGAGTC
Zip11	CTTCTTCACCTGGGCAATGT	GGAGGTCAGCCAGGTAGACA
Zip13	TGCCTGTCGCCTGGATAATAA	ACTGAGCCCAACCATGAGAGA
Zip14	CGCCATTGAAGTATGGGGGTA	GCAGGAGCCTCTTGTAAAAAGT
Znt1	GCTCTCGAGTTGGTCCTGTC	GCCTCATGGTGAGGTAGGAA
Znt3	CCATCAGCACCTTCCTCTTC	ATGGAGATCATGGGTTGCTC
Znt4	TGCCGTCCTCTACTTGCTTT	TAGGCGATGAAATCCAAAGG
Znt5	TTGGTTTTCATACGGCTTCC	TTTGGACACGTCCATTTTGA
Znt6	ATGGGGACGATTCATCTCTTTCG	CACAGCACGTTGATTGCACC
Znt7	GGATGATGAATACAAACCACCCA	AAAGCGAAAGAGAGGTTCAGG
Znt9	CCAGGATGTTTCCGGGCTT	GCTCTGTCTGGGTTGGAGA

Table S3. Detailed time for the western blot membrane trimming workflow.

Figure	Antibody	Exposure date	Exposure times
1E	DDX4	24.5.29	10s
1E	β-actin	24.5.29	3s
2E	ZIP6	24.8.29	30s
2E	β-actin	24.8.29	10s
3C	ATF4 (12 h)	24.12.20	10s
3C	NRF2 (12 h)	25.12.20	10s
3C	γ-H2AX (12 h)	24.12.25	10s
3C	p-ERK1/2 (12 h)	24.12.27	10s
3C	ERK1/2 (12 h)	24.12.27	15s
3C	β-actin (12 h)	25.12.20	3s
3C	ATF4 (24 h)	24.9.22	10s
3C	NRF2 (24 h)	24.9.22	10s
3C	γH2AX (24 h)	24.9.20	10s
3C	p-ERK1/2 (24 h)	24.12.20	10s
3C	ERK1/2 (24 h)	24.12.20	15s
3C	β-actin (24 h)	25.9.22	3s
4C	LAMP1 (12 h)	25.1.12	10s
4C	p62 (12 h)	24.11.4	10s
4C	LC3B (12 h)	24.11.7	20s
4C	β-actin (12 h)	24.11.7	3s
4C	LAMP1 (24 h)	25.12.25	10s
4C	p62 (24 h)	24.9.19	10s
4C	LC3B (24 h)	24.9.19	20s
4C	β-actin (24 h)	24.9.19	10s
5E	BAX (12 h)	24.10.22	15s
5E	BCL2 (12 h)	24.10.22	10s
5E	Cleaved Caspase 3 (12 h)	24.10.22	30s
5E	β-actin (12 h)	25.10.22	3s
5E	BAX (24 h)	24.9.26	15s
5E	BCL2 (24 h)	24.9.26	10s
5E	Cleaved Caspase 3 (24 h)	24.5.31	30s
5E	β-actin (24 h)	24.9.26	3s
7C	ZIP6	25.2.2	30s
7C	β-actin	25.2.2	3s
7H	LAMP1	25.2.18	10s
7H	p62	25.2.18	10s
7H	LC3B	25.2.19	20s
7H	β-actin	25.2.18	3s
7H	γH2AX	24.10.29	15s
7H	BAX	24.11.2	15s
7H	BCL2	24.11.2	10s
7H	Cleaved Caspase 3	24.10.30	30s
7H	β-actin	24.10.30	3s
