# Supplementary material for: Granulosa Cell-Secreted KITL Is Involved in Maintaining Zinc Homeostasis in the Oocytes of Neonatal Mouse Ovaries
Source: Antioxidants (Basel). 2025 Nov 10;14(11):1345. doi: 10.3390/antiox14111345 (PMC12649686; doi:10.3390/antiox14111345)
Supplement: Supplementary file 1 [file antioxidants-14-01345-s001.zip › Western Blot Data Integrity Statement.pdf]

Western Blot Data Integrity Statement

To optimize the use of precious in vivo samples and adhere to the 3Rs principles (Replacement, Reduction, Refinement), the nitrocellulose membranes were strategically trimmed with a clean scalpel to isolate the relevant molecular weight ranges for the targets of interest before the probing procedure. This approach precluded the need for stripping and re-probing, which can compromise data quality.

Regarding the presentation of the western blot data:

1. We affirm that all blot images presented in the figures are direct, continuous scans of the membrane sections. No image splicing, re-arrangement of lanes, or deletion of any data lanes has been performed.
2. Any post-acquisition image processing was restricted to global adjustments of brightness and/or contrast, applied uniformly to the entire image to improve visual clarity, without altering the underlying data interpretation.
